# Assessment of immediate effects of percutaneous balloon mitral valvuloplasty on right ventricular and pulmonary functions in severe rheumatic mitral stenosis patients using speckle tracking echocardiography and spirometry

**DOI:** 10.34172/jcvtr.2020.46

**Published:** 2020-11-24

**Authors:** Praveen Kumar Reddy Sakkuru, Vanajakshamma Velam, Rajasekhar Durgaprasad, Narendra Chanda, Raja Naga Mahesh Maddala, Madhava Naidu Yandrapu

**Affiliations:** ^1^Department of Cardiology, Sri Venkateswara Institute of Medical Sciences, Tirupati, Andhra Pradesh, India

**Keywords:** Mitral Stenosis, Percutaneous Balloon Mitral Valvuloplasty, Right Ventricle, Speckle Tracking Echocardiography, Pulmonary Function

## Abstract

***Introduction:*** The current study was sought to assess the immediate effect of percutaneous balloonmitral valvuloplasty (PBMV) on right ventricular (RV) and pulmonary functions using speckle tracking echocardiography (STE) and spirometry respectively.

***Methods:*** Two-dimensional speckle tracking and doppler studies for strain and strain rate imaging of RV were performed before PBMV, after 48h and 15 days of PBMV using echocardiography and spirometry. Mitral valve area, peak and mean mitral valve transannular pressure gradients, late filling velocities,Wilkins score, Systolic pulmonary artery pressure, TAPSE, RV end-diastolic and end-systolic areas,RV fractional area change and Tei index were measured.

***Results:*** There was a significant rise in peak RV global longitudinal strain (GLS) from baseline to48h post PBMV and at 15 days post PBMV. Segmental RV strain at basal septum, mid septum,apical septum and basal RV free wall showed considerable improvement from baseline to 48h post PBMV and 15 days post PBMV. RV longitudinal strain rate parameters did not show significant improvement after PBMV and remained low at follow-up. Post PBMV all patients showed restrictive features on pulmonary function test. The mean FEV_1_ (% predicted), mean FVC (% predicted), mean PEFR improved from baseline to 48h PBMV and 15 days post PBMV. Though the mean FEV_1_/FVC increased post PBMV at 15 days follow-up, but it was statistically insignificant.

***Conclusion:*** RV performance in MS was decreased mainly due to increase in RV after load which improves after PBMV. Patients with severe MS have impaired pulmonary function which is of restrictive type and successful PBMV improves pulmonary function.

## Introduction

Rheumatic heart disease (RHD) is one of the noticeable causes of mortality and morbidity in developing countries across the globe. It has been estimated that 15.6 million people are affected by RHD globally.^1^ Every year, approximately 470 000 new cases and 233 000 deaths were attributed due to RHD.^2^ RHD is a major cardiac problem in India. The prevalence rates have been found to be 0.2 to 1.1/1000 populations.^3^ Rheumatic mitral stenosis (MS) is the most common RHD which includes characteristic changes of the mitral valve include thickening at the leaﬂet edges, fusion of the commissures, chordal shortening and fusion.^4^ Subsequent scarring leads to valve deformity resulting in a small fish mouth orifice.^5^

Long standing pulmonary arterial hypertension can result in morphological changes in the pulmonary vasculature comprising of endothelial proliferation and medial hypertrophy. This causes secondary right ventricular (RV) overload, dilatation and failure. Pulmonary dysfunction in severe MS is attributed to interstitial and alveolar edema, reactive fibrosis, pleural effusion and decreased lung volumes.^6^

Abnormalities in RV function play vital role in the development of clinical symptoms and prognosis of MS patients.^7^ Among many indices, strain and strain rate are novel to assess RV function. For the evaluation of RV function by strain and strain rate, Tissue Doppler imaging (TDI) is used.^8^ A new method, Two-dimensional speckle tracking, to quantify strain and strain rate for assessment of global and regional myocardial function. Lack of angle dependency is the main advantage of strain derived by speckle tracking over strain derived by TDI, which may result in more reliable strain measurements.

There are studies on long-term improvement in RV and pulmonary functions in patients with MS after percutaneous balloon mitral valvuloplasty (PBMV)^9^ whereas only few studies examined the immediate effect of PBMV on RV function using speckle tracking echocardiography (STE).^10^ The current study was sought to assess the immediate effect of PBMV on RV and pulmonary functions using STE and spirometry respectively.

## Materials and Methods

This prospective, observational study was conducted between May 2017 and December 2018 which consisted of 52 patients with symptomatic severe Rheumatic mitral stenosis. This study was approved by the institutional ethics committee of our institute [IEC number: 639]. A written informed consent was obtained from all the study participants.

### Inclusion criteria

1. Patients aged >18 years.

2. Patients with severe MS (MVA<1.0 cm²).

3. Patients with successful PBMV.

### Exclusion criteria

1. Patients having severe aortic valve disease associated with mitral stenosis.

2. Patients with atrial fibrillation, coronary heart disease, congenital heart disease, mitral regurgitation (MR) grade more than 2+ after PBMV.

3. Patients with diabetes and hypertension.

4. Pregnant women.

5. Patients who refused to give consent.

### Data Collection

Baseline clinical and demographic characteristics were obtained from all patients. Two-dimensional speckle tracking and doppler studies for strain and strain rate imaging of RV were performed before and 48h after PBMV using American Society of Echocardiography recommendations^11^ and spirometry using American Thoracic Society recommendations.^12^ Echocardiography was performed by using SIEMENS ACUSON S2000 echocardiography machine with 3.5MHz probe. Spirometry was performed by using Easy One Pro^TM^Lab, ndd Medical Technologies Andover, MA, USA.

Mitral valve area (MVA) was determined by planimetry. The peak and mean mitral valve transannular pressure gradients and late filling velocities were measured using continuous wave doppler recordings through mitral inflow. Wilkins score^13^ was used to judge mitral leaflet mobility, valvular and subvalvular thickening, and calcification. Systolic pulmonary artery pressure was derived from the tricuspid regurgitant jet peak velocity using the modified Bernoulli equation. The tricuspid annular plane systolic excursion (TAPSE) was determined by the difference in the displacement of the RV base during systole and diastole. RV end diastolic and end-systolic areas were measured from the apical four chamber view to calculate RV fractional area change (RVFAC). The Tei index of RV myocardial performance was calculated as the time between tricuspid valve closure to tricuspid valve opening, divided by the RV ejection time, determined by pulsed Doppler.

Two-dimensional images were acquired from the four-chamber view for offline analysis using the two-dimensional strain software. The endocardial borders of the RV were traced manually by point and click approach and tracked by the software. An epicardial surface tracing is then automatically generated by the system. After manual adjustment of the region of interest, software automatically divides the region of interest into 6 segments. The RV free wall and interventricular septum were divided in three segments, basal, mid, and apical, for quantification of regional systolic strain. The GLS was calculated for the entire the right ventricle.^14^

Spirometry was performed to evaluate the lung function disturbances, according to the recommendations of American Thoracic Society.^12^ Patients were instructed to inhale as much as possible and then exhale rapidly and forcefully for as long as flow is maintained. Patient should exhale for at least six seconds. At the end of the forced exhalation, the patient should again inhale fully as rapidly as possible. The following parameters were determined -- Forced vital capacity (FVC), forced expiratory volume in first second (FEV_1_), FEV_1_/FVC and peak expiratory flow rate (PEFR) rate. Interpretation of pulmonary function tests: Restriction was defined as FVC< 80% of predicted with normal or increased FEV_1_/FVC.^15^ Obstruction was defined low FEV_1_/FVC ratio (<0.7) and FEV_1_<80% predicted.^16^ Mixed restriction and obstruction is defined as low FVC (<80% predicted), FEV_1_ (<80% predicted) along with reduced FEV_1_/FVC ratio (<0.7).^16^

Echocardiography was repeated 48 h after the procedure to evaluate the final MVA and to assess the degree of residual MR. Successful PBMV was defined as post valvuloplasty MVA >1.5 cm^2^ with no more than 2+ MR. Two-dimensional (2D) speckle tracking, Doppler studies were repeated after 2 weeks to assess immediate changes in parameters. Pulmonary function tests were repeated after 2 weeks to assess immediate changes.

### Statistical analysis

Continuous variables were expressed as mean and standard deviation (SD). Paired Student’s t-test was used to determine the significance among the parameters pre and post PBMV. Statistical Package for Social Sciences (SPSS) version 20.0 (IBM Corp, Somers, NY, USA) was used for the analysis. A *P* value of ≤0.05 was considered as significant.

## Results

This study findings were conceived from 52 symptomatic severe rheumatic MS patients who underwent PBMV. Mean age of the study population was 35.8±9.2 years. Majority of them were female patients (72%). Dyspnea was the presenting symptom in all the cases. Sixty one percent were in NYHA class-III and 39% were in NYHA class-II. Six patients (11%) had undergone prior PBMV and 2 patients (4%) had undergone prior closed mitral valvotomy (CMV). The mean Wilkins score was 6.5±1.2. Demographic and clinical characteristics of the study population at baseline were shown in Table 1.

**Table 1:** Baseline clinical and demographic patient characteristics

**Characteristic**	**Mean±SD / n (%)**
Age (years)	35.8±9
Sex
Women	38 (73%)
Men	14 (27%)
NYHA Functional class
NYHA class II	21 (39%)
NYHA class III	31 (61%)
CVA history	2 (4%)
History of previous PBMV	6 (11%)
History of previous CMV	2 (4%)
Wilkins score	6.5±1.2

Abbreviations: SD, standard deviation; NYHA, *New York Heart Association;* CVA, cerebrovascular accident; PBMV, percutaneous balloon mitral valvuloplasty; CMV, closed mitral valvotomy

### Conventional echocardiographic parameters

Comparison of baseline and follow-up echocardiographic characteristics were summarized in Table 2. PBMV was generated a significant increase in mean MVA, TAPSE, FAC and a significant decrease in peak mitral valve gradient, mean mitral valve gradient, pulmonary artery systolic pressure (PASP) from baseline to 48 hours post PBMV and 15 days post PBMV. There was an insignificant increase in RV MPI at 48 hours post PBMV with lack of considerable improvement at 15 days post PBMV.

**Table 2:** Comparison of baseline and follow-up conventional echocardiographic characteristics

**Parameter**	**Pre PBMV** **(A)**	**Post PBMV 48 hours (B)**	***P *** **value** **(A vs B)**	**Post PBMV 15 days (C)**	***P *** **value** **(A vs C)**
2D MVA (cm^2^)	0.73 ± 0.27	1.73 ± 0.29	<0.001*	1.81 ± 0.13	<0.001*
Peak MVG (mm Hg)	26.7±7.9	9.6±2.8	<0.001*	8.2±2.8	<0.001*
Mean MVG (mm Hg)	15.9±4.5	4.6±1.1	<0.001*	4.2±1.5	<0.001*
PASP (mm Hg)	56.8±20.3	45.5±15.2	<0.001*	38.9±15.7	<0.001*
TAPSE (mm)	19.21±4.13	23.84±7.61	0.015*	51.51±11.8	0.008*
FAC (%)	36.71±15.76	48.85±11.32	<0.001*	51.51±11.8	<0.001*
RV MPI	0.71±0.15	0.72±0.12	0.256	0.73±0.21	0.162

Abbreviations: PBMV, percutaneous balloon mitral valvuloplasty; MVA, mitral valve area; PASP, pulmonary artery systolic pressure; MVG, mitral valve gradient; TAPSE, tricuspid annular plane systolic excursion; FAC, fractional area change; RV MPI, right ventricular myocardial performance index Paired Student’s *t* test. *indicates significant *P* value (*P*≤0.05)

### Speckle tracking echocardiographic parameters

**Figure 1:**
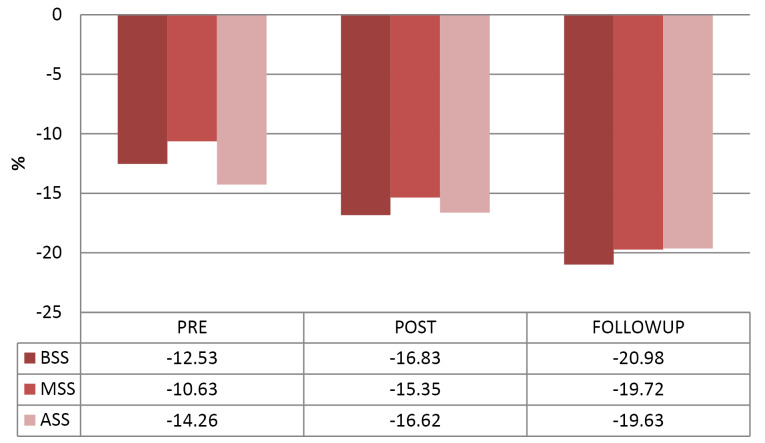
Comparison of longitudinal strain in IVS from baseline to follow-ups

BSS: Basal septum strain; MSS: Mid septum strain; ASS: Apical septum strain; PRE: Baseline; POST: Post PBMV 48 hours; FOLLOW UP: Post PBMV 15 days

**Figure 2:**
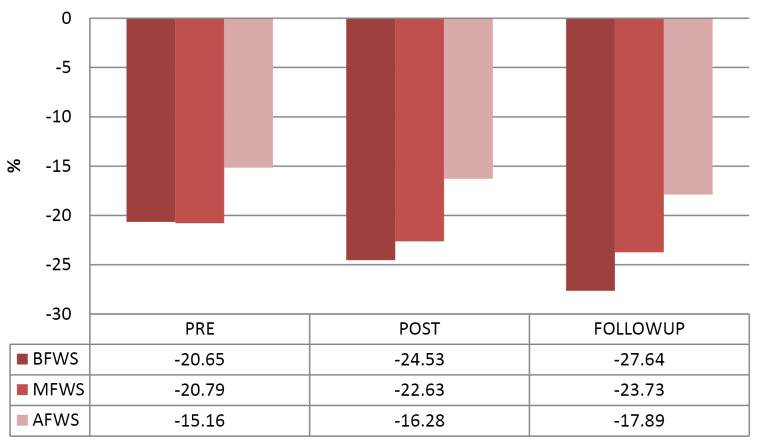
Comparison of longitudinal strain of RV free wall from baseline to follow-ups

BFWS: Basal free wall strain, MFWS: Mid free wall strain, AFWS: Apical free wall strain; PRE: Baseline; POS: Post PBMV 48 hours; FOLLOW UP: Post PBMV 15 days

**Figure 3:**
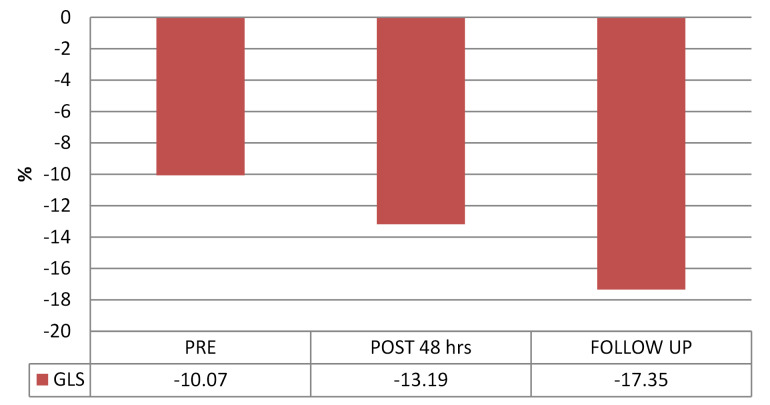
Comparison of RV global longitudinal strain from baseline to follow up

PRE: Baseline; POST: Post PBMV 48 hours; FOLLOW UP: Post PBMV 15 days; GLS: Global longitudinal strain

Comparison of baseline and follow-up RV longitudinal strain parameters were shown in Table 3, Figure 1, Figure 2 and Figure 3. There was a significant rise in peak RV GLS from baseline to 48 hours post PBMV and at 15 days post PBMV follow-up. Segmental RV strain at basal, mid and apical septum and basal RV free wall showed considerable improvement from baseline to 48 hours post PBMV and 15 days post PBMV. No significant improvement was observed in segmental RV strain at mid RV free wall, apical free wall strain at 48 hours post PBMV and 15 days post PBMV.

**Table 3:** Comparison of baseline and follow-up RV longitudinal strain parameters

**Longitudinal** **strain (%)**	**Pre PBMV** **(A)**	**Post PBMV 48 hours (B)**	***P *** **value** **(A vs B)**	**Post PBMV 15 days (C)**	***P *** **value** **(A vs C)**
Basal septum	-12.53±7.43	-16.83±6.31	0.011*	-20.98 ±7.39	0.008*
Mid septum	-10.63±6.68	-15.35±7.89	0.015*	-19.72±5.26	0.003*
Apical septum	-14.26±6.37	-16.62±5.73	0.017*	-19.63±7.81	0.002*
Basal RV FW	-20.65±12.27	-24.53±11.97	0.014*	-27.64±12.58	0.023*
Mid RV FW	-20.79±9.37	-22.63±10.42	0.091	-23.73±11.74	0.058
Apical RV FW	-15.16±8.91	-16.28±9.87	0.061	-17.89±10.78	0.069
Global RV	-10.07±4.70	-13.19±4.18	0.011*	-17.35±4.54	0.009*

Abbreviations: PBMV, percutaneous balloon mitral valvuloplasty; RVFW, Right ventricle free wall; RV, Right ventricle Paired Student’s *t* test. *indicates significant *P*value (*P*≤0.05)

In our study RV longitudinal strain rate parameters did not show significant improvement after PBMV and remained low at follow-up. Details of RV longitudinal strain rate parameters at baseline, post PBMV 48 hours and post PBMV 15 days follow up were summarized in Table 4.

**Table 4:** Comparison of baseline and follow up RV longitudinal strain rate parameters.

**Strain rate (S⁻¹)**	**Pre PBMV** **(A)**	**Post PBMV 48 hours (B)**	***P*** ** value** **(A vs B)**	**Post PBMV 15 days (C)**	***P*** ** value** **(A vs C)**
Basal septum	-1.18±0.79	-1.20±.0.99	0.623	-1.22±0.86	0.568
Mid septum	-1.09±1.33	-1.11±1.42	0.551	-1.06±0.89	0.492
Apical septum	-0.99±0.48	-1.02±0.86	0.359	-1.03±0.59	0.321
Basal RV FW	-2.87±1.87	-3.01±1.96	0.456	-3.21±2.12	0.412
Mid RV FW	-3.31±2.17	-3.09±1.87	0.562	-3.12±1.98	0.608
Apical RV FW	-1.53±1.42	-1.44±1.51	0.484	-1.49±1.15	0.521

Abbreviations: RV FW, Right ventricle free wall. Paired Student’s *t* test. *indicates significant *P* value (*P*≤0.05).

### Pulmonary function parameters

In our study 4 patients (7%) had obstructive airway disease and remaining of the 48 patients (93%) had restrictive features on pulmonary function test before PBMV. Post PBMV all patients showed restrictive features on pulmonary function test. The mean FEV_1_ (% predicted), mean FVC (% predicted), mean PEFR improved from baseline to 48 hours PBMV and 15 days post PBMV. Though the mean FEV_1_/FVC (108.52±17.21) increased post PBMV at 15 days follow-up, but it was statistically insignificant (Table 5).

**Table 5:** Changes in pulmonary function test before PBMV and 15 days after PBMV.

**Parameter**	**Before PBMV**	**Post PBMV 15 days**	***P*** ** value**
FEV_1_ (%predicted)	62.18 ± 11.05	75.32 ± 11.67	<0.001*
FVC (%predicted)	54.80± 11.31	69.57 ± 12.66	<0.001*
FEV_1_/FVC (%)	108.52±17.21	110.45±13.25	0.351
PEFR (L/min)	229.87±68.59	342.08±59.39	<0.001*

Abbreviations: FEV_1_, Forced expiratory volume at 1 second; FVC, Forced vital capacity; PEFR, Peak expiratory flow rate. Paired Student’s *t* test. *indicates significant p-value (*P*≤0.05).

## Discussion

In our study there was a significant increase in 2D MVA, FAC, TAPSE at 48 hours following PBMV which was maintained post PBMV at 15 days follow-up. We observed a significant decrease in PASP, mean mitral valve gradient and peak mitral valve gradient. Similar findings were observed in studies done by Roushdy et al^17^, Alabaady M et al^18^ and Kumar V et al^19^. These findings suggest immediate improvement in RV function following PBMV. Conventionally, for evaluation of procedural success, these echocardiographic parameters are valuable.

In our study RV MPI showed no significant change after PBMV at 48 hours and at 15 days follow-up which shows that immediate improvement of RV function is due to decreased after load of RV. RV MPI is a load independent parameter of RV function. Similar observations were reported by Mohan et al.^7^

In our study there was significant rise in RV GLS at 48 hours after PBMV and at 15 days follow-up. Also found a significant rise in RV segmental strain at basal septum of IVS, mid septum of IVS, apical septum of IVS, basal RV free wall at 48 hours post PBMV and at 15 days follow-up. Statistically insignificant difference was observed in RV segmental strain at mid RV free wall and apical RV free wall at 48 hours post PBMV and lack of improvement at 15 days follow-up. Similar findings were reported by Kumar V et al^19^ and Alabaady M et al^18^. Enhanced LV filling and LV contractility following PBMV may be the reasons for improvement of IVS strain than RV free wall strain in our study.

We observed no change in RV segmental strain rates in any of the RV segments. Similar findings were reported by Roushdy et al and Kumar V et al.^17,19^ Strain rate is more affected by contractility and is not affected by loading conditions. This means that increased afterload is the main cause of RV systolic dysfunction in severe MS patients. In our study RV GLS has strong correlation with PASP than other conventional echocardiographic parameters. Similar observations were made in previous studies by Roushdy MA et al, Alabaady M et al, and Kumar V et al.^17-19^

In our study 4 patients (7%) had obstructive type airway disease and remaining 48 patients (93%) had restrictive type of features on pulmonary function test before PBMV. Post PBMV all patients showed restrictive type of features on pulmonary function test. Similar findings were reported by Simkova et al and Khan et al.^20,21^ The restrictive features may be due to chronic lung congestion due to increased interstitial fluid, decreased lung compliance and fibrosis from chronic congestion and consequent muscle fatigue.

In our study, 15 days post PBMV, there was significant improvement in pulmonary function parameters FEV_1_ (%predicted), FVC (%predicted) and PEFR. However, the changes in FEV1/FVC were statistically insignificant. Similar observations were reported by Khan et al and Mundhra SH et al.^21,22^ All the patients showed improvement of their ventilatory function after PBMV that may be due to reduction in left atrial hypertension, pulmonary venous congestion, resulting in increased lung compliance.

The number of patients studied were relatively less and study was done in severe MS patients only. Short axis images of RV were not recorded. Doppler strain was not included for comparison. Short duration of follow-up.

## Conclusion

Right ventricular function can be assessed by global and segmental RV strain using STE comparable with conventional echocardiographic parameters. RV performance in MS is reduced mainly due to rise in RV afterload which improves after PBMV. Patients with severe MS have impaired pulmonary function which is of restrictive type and successful PBMV improves pulmonary function.

## Competing interests

None.

## Ethical approval

This study was conducted with a prior approval from the institutional ethics committee (IEC number: 639) and an informed consent was obtained from all the study participants prior to the enrolment.

## Funding

None.

## References

[1] Carapetis JR, McDonald M, Wilson NJ (2005). Acute rheumatic fever. Lancet.

[2] WHO Study Group on Rheumatic Fever and Rheumatic Heart Disease, World Health Organization. Rheumatic Fever and Rheumatic Heart Disease: Report of a WHO Expert Consultation, Geneva, 20 October - 1 November 2001. WHO; 2004.

[3] Shah B, Sharma M, Kumar R, Brahmadathan KN, Abraham VJ, Tandon R (2013). Rheumatic heart disease: progress and challenges in India. Indian J Pediatr.

[4] Roy SB, Gopinath N (1968). Mitral stenosis. Circulation.

[5] Tsang W, Freed BH, Lang RM. Three-dimensional anatomy of the aortic and mitral valves. In: Otto CM, Bonow RO, eds. Valvular Heart Disease: A Companion to Braunwald’s Heart Disease. 4th Ed. Philadelphia: Saunders; 2013. p. 14-29.

[6] Braunwald Eugene. Valvular heart disease. In: Braunwald Eugene. Heart disease: A textbook of Cardiovascular Medicine. 5th ed. Philadelphia: W.B. Saunders; 1997. p. 1007-1016.

[7] Mohan JC, Sengupta PP, Arora R (1999). Immediate and delayed effects of successful percutaneous transvenous mitral commissurotomy on global right ventricular function in patients with isolated mitral stenosis. Int J Cardiol.

[8] Kukulski T, Hübbert L, Arnold M, Wranne B, Hatle L, Sutherland GR (2000). Normal regional right ventricular function and its change with age: a Doppler myocardial imaging study. J Am Soc Echocardiogr.

[9] Hirata N, Sakakibara T, Shimazaki Y, Watanabe S, Nomura F, Akamatsu H (1992). Preoperative and postoperative right ventricular function during exercise in patients with mitral stenosis. J Thorac Cardiovasc Surg.

[10] Pavlopoulos H, Nihoyannopoulos P (2008). Strain and strain rate deformation parameters: from tissue Doppler to 2D speckle tracking. Int J Cardiovasc Imaging.

[11] Schiller NB, Shah PM, Crawford M, DeMaria A, Devereux R, Feigenbaum H (1989). Recommendations for quantitation of the left ventricle by two-dimensional echocardiography American Society of Echocardiography Committee on Standards, Subcommittee on Quantitation of Two-Dimensional Echocardiograms. J Am Soc Echocardiogr.

[12] Standardization of spirometry, 1994 update (1995). American Thoracic Society. Am J Respir Crit Care Med.

[13] Wilkins GT, Weyman AE, Abascal VM, Block PC, Palacios IF (1988). Percutaneous balloon dilatation of the mitral valve: an analysis of echocardiographic variables related to outcome and the mechanism of dilatation. Br Heart J.

[14] Leitman M, Lysyansky P, Sidenko S, Shir V, Peleg E, Binenbaum M (2004). Two-dimensional strain-a novel software for real-time quantitative echocardiographic assessment of myocardial function. J Am Soc Echocardiogr.

[15] Suresh V, Reddy A, Mohan A, Rajgopal G, Satish P, Harinarayan C (2011). High prevalence of spirometric abnormalities in patients with type 1 diabetes mellitus. Pediatr Endocrinol Diabetes Metab.

[16] (1991). Lung function testing: selection of reference values and interpretative strategies. American Thoracic Society. Am Rev Respir Dis.

[17] Roushdy AM, Raafat SS, Shams KA, El-Sayed MH (2016). Immediate and short-term effect of balloon mitral valvuloplasty on global and regional biventricular function: a two-dimensional strain echocardiographic study. Eur Heart J Cardiovasc Imaging.

[18] Alabaady M, Ali A, Alsawasany M, Al-Deftar M, Swailum AS (2017). Immediate and short-term effect of balloon mitral valvuloplasty on circumferential strain, global and regional biventricular systolic function: two dimensional strain echocardiographic study. Researcher.

[19] Kumar V, Jose VJ, Pati PK, Jose J (2014). Assessment of right ventricular strain and strain rate in patients with severe mitral stenosis before and after balloon mitral valvuloplasty. Indian Heart J.

[20] Simkova I, Urbanova J (2001). Pulmonary function alterations after correction of mitral stenosis. BratislLekListy.

[21] Khan US, Islam AM, Majumder AA (2015). TCTAP A-120 effect of successful percutaneous transvenous mitral commissurotomy for mitral stenosis on pulmonary function. J Am Coll Cardiol.

[22] Mundhra SH, Mundhara KS, Thakkar RM, Ninama K, Parmar H (2015). Pulmonary function test in mitral valve disease. Int Arch Integr Med.

